# High Quality ATAC-Seq Data Recovered from Cryopreserved Breast Cell Lines and Tissue

**DOI:** 10.1038/s41598-018-36927-7

**Published:** 2019-01-24

**Authors:** Saori Fujiwara, Songjoon Baek, Lyuba Varticovski, Sohyoung Kim, Gordon L. Hager

**Affiliations:** Laboratory of Receptor Biology and Gene Expression, National Cancer Institute, National Institutes of Health, Bethesda, MD 20892 USA

## Abstract

DNA accessibility to transcription regulators varies between cells and modulates gene expression patterns. Several “open” chromatin profiling methods that provide valuable insight into the activity of these regulatory regions have been developed. However, their application to clinical samples has been limited despite the discovery that the Analysis of Transposase-Accessible Chromatin followed by sequencing (ATAC-seq) method can be performed using fewer cells than other techniques. Obtaining fresh rather than stored samples and a lack of adequate optimization and quality controls are major barriers to ATAC’s clinical implementation. Here, we describe an optimized ATAC protocol in which we varied nuclear preparation conditions and transposase concentrations and applied rigorous quality control measures before testing fresh, flash frozen, and cryopreserved breast cells and tissue. We obtained high quality data from small cell number. Furthermore, the genomic distribution of sequencing reads, their enrichment at transcription start sites, and transcription factor footprint analyses were similar between cryopreserved and fresh samples. This updated method is applicable to clinical samples, including cells from fine needle aspiration and tissues obtained via core needle biopsy or surgery. Chromatin accessibility analysis using patient samples will greatly expand the range of translational research and personalized medicine by identification of clinically-relevant epigenetic features.

## Introduction

Despite sharing the same genetic information, cells from distinct tissues and lineages often display vastly different gene expression profiles. A key modulator of this phenomenon is how DNA is packaged by histone proteins, known as chromatin, in different cells. When chromatin is in a relaxed state, or “open”, transcription start sites, and nearby regulatory promoter and enhancer regions are accessible to transcription factors and other enzymes that modulate transcription. Open chromatin can be identified by DNA cleavage using several enzymes, such as deoxyribonuclease I (DNase I)^1^ and micrococcal nuclease^2^, or by sonication^3^. The genome-wide signature of these hypersensitive open chromatin sites may predict disease status and response to therapy^4–6^. Within open chromatin sites, narrow sites of protection (footprints) correlate with transcription factors bound to their recognition motifs. Using this information, we previously published a “digital genomic footprinting” analysis that characterizes these regulatory sites^7^. When applied to breast cancer cells and mouse liver tissue, this analysis identifies active regulatory sites, specific transcription factor occupancies, and networks^8–10^. Therefore, identifying open chromatin sites by hypersensitivity to enzymatic or physical cleavage is a powerful approach to annotate regulatory regions of genomes associated with disease status and provides valuable information for developing novel diagnostic and therapeutic strategies.

However, the DNA hypersensitivity methods mentioned above require millions of cells, which represents a major obstacle to performing global chromatin landscape analysis using clinical samples, which typically contain less than 100,000 cells. Samples obtained during open surgery or by biopsy are also commonly fixed in formalin or frozen for later analysis of tissue sections or other means. Fortunately, the Analysis of open chromatin sites by Transposase-Accessible Chromatin followed by deep sequencing (ATAC-seq) hypersensitivity method was recently reported to only require small number of fresh cells^11–13^. After nuclei are isolated from samples, this assay uses hyperactive Tn5 transposase^14^ to simultaneously cut and ligate specific sequences at accessible chromatin regions. The transposase reaction, called “tagmentation”, allows for amplification of DNA fragments by PCR and the amplified DNA are suitable for high-throughput sequencing. The ATAC-seq has also been used on stored, slowly frozen human embryonic stem cells^15^ and hematopoietic B cells^16^, and a modified protocol was developed for flash-frozen human thyroid cancer and brain tissues (Omni-ATAC)^17^. However, previous studies did not provide adequate data to assist researchers who wish to optimize the ATAC-seq protocol for other samples, including a comparison between fresh and frozen stored cells or tissues, which is critical information needed to optimize this method for other clinical applications.

In this study, we optimized the ATAC-seq method for fresh and cryopreserved breast cancer cells and mouse mammary tissues. We found that high quality genome-wide open chromatin landscape data that is comparable to that produced using living cells can be generated from a small number of cells, as well as from small tissue samples stored by cryopreservation. Adapting ATAC-seq to small, stored clinical samples will greatly expand the reach of translational research and allow researchers to fully characterize the link between chromatin landscape changes and disease. This approach may also be applicable for personalized medicine therapeutic strategies employed by clinicians treating breast cancer and other diseases.

## Results

### ATAC protocol optimization for human breast cancer cell lines

**Figure 1:**
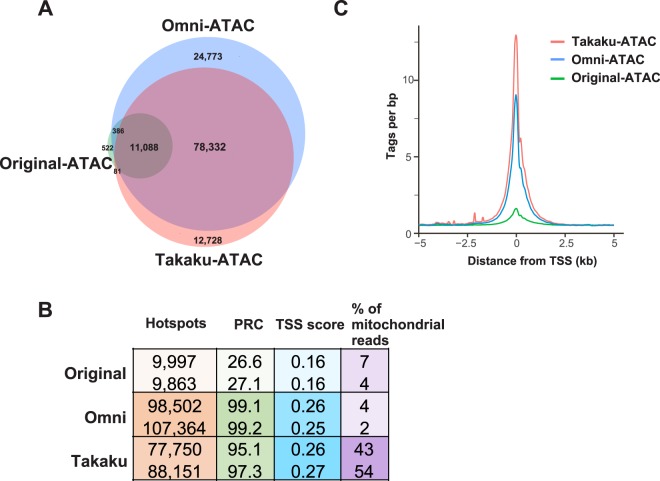
Comparison of three ATAC protocols using 50,000 fresh MCF7 cells. (**A**) Venn diagram showing the total number of common and unique hot spots from two merged replicates using original ATAC (green), Omni-ATAC (blue) and Takaku-ATAC (red) protocols. (**B**) Table showing total hot spot count, PRC, TSS score and % mitochondrial reads for individual replicates to compare quality and verify reproducibility of each protocol. (**C**) Histogram of tag count enrichment reads at TSS +/−5 kb for merged replicates using each protocol. Original ATAC (green), Omni-ATAC (blue) and Takaku-ATAC (red) protocols.

The ATAC protocol is reported to require optimization for each cell type and tissue^18,19^. The first step of ATAC procedure is nuclear preparation from samples. Thus, we firstly compared three previously published nuclear preparation step using different cell lysis buffers from 50,000 live MCF7 cells. We assessed data quality using the original ATAC protocol lysis buffer (0.1% NP40)^12^, the Omni-ATAC protocol lysis buffer (0.1% NP40, 0.1% Tween-20 and 0.01% Digitonin)^17^, and the Takaku-ATAC protocol (0.1% Triton X-100)^18^. Nuclei preparation efficiency was estimated by trypan blue exclusion and 20%, 45%, and 99% of cells were found to be permeabilized using the original, Omni, and Takaku protocols, respectively. However, the Omni protocol nuclei yield reached 90% upon the addition of 0.1%Tween-20 and 0.01% Digitonin during the following transposase reaction step. Similar sequencing data were obtained by the Takaku and Omni protocols, including total tag count (Fig. 1A) and peak distributions, also referred as genomic distribution hot spots (Supplementary Fig. 1). In contrast, the original ATAC-seq method produced a relatively small number of hotspots (Fig. 1A,B, Supplementary Fig. 1B), with a significantly higher fraction of hot spots located in promoters (Supplementary Fig. 1A). To evaluate the signal-to-noise ratio and to compare the efficiency of the transposase reaction, we used the Transcription Start Site enrichment score (TSS score), as recommended by ENCODE, and the Percent Reference peak Coverage (PRC) metric developed in our laboratory to ensure compatible levels of digestion (Fig. 1B). PRC is a rate of hotspots overlapping with common open chromatin sites of the species and can evaluate hotspots distant from TSS. Both TSS and PRC scores were similar between the Takaku and Omni ATAC-seq protocols, but significantly better than the data obtained using the original ATAC protocol. Although, the Takaku protocol resulted in a higher proportion of mitochondrial reads, it also had the best TSS read counts (Fig. 1C). Thus, we conclude that inefficient nuclear preparation obtained using the original ATAC protocol resulted in lower number of peaks, lower PRC, and a higher promoter-localized fraction. In addition, the Takaku protocol is simpler and has fewer manipulation steps, which will avoid DNA loss steps and thus beneficial for clinical laboratories handling small samples.

The nuclear preparation step is followed by tagmentation using transposase. The optimal transposase concentration is essential for high quality of ATAC-seq data. Thus, we further optimized the Takaku protocol using fresh MCF7 cells by increasing the transposase concentration from 2X to 4X compared to previously published ATAC protocol^12^, keeping the total volume of the reaction constant at 50 µl. The higher transposase concentration improved genome coverage, produced higher total hot spot numbers, and a lower signal-to-noise ratio without affecting the fraction of hot spots in promoters (Supplementary Fig. 2). Hereafter, we refer to the optimized protocol as OPTI-ATAC. We confirmed that OPTI-ATAC was also suitable and significantly better than the original ATAC-seq protocol when applied to other breast cancer cell lines (T47D and ZR75-1, Supplementary Fig. 3).

We then tested whether the OPTI-ATAC protocol can be applied to smaller cell number samples (10,000, 25,000 cells). The total reaction volume for 10,000 cells was decreased to 20 µL and the transposase concentration was increased to 10X. Under these conditions, OPTI-ATAC performed using 10,000 cells produced a similar percentage of uniquely mapped reads and was almost identical to OPTI-ATAC using 50,000 cells across number of hotspots, PRC, TSS score and rate of mitochondrial reads (Supplementary Fig. 4 and Table 1).

**Table 1:** Total hot spot count, PRC, TSS score and % mitochondrial reads for individual replicate was calculated from OPTI-ATAC-seq performed on 10,000 and 50,000 fresh, as well as 10,000 cryopreserved and 50,000 flash frozen MCF7 cells.

ATAC method	Storage method	Number of cells	Replicate	Hotspots	PRC	TSS score	% mitochondrial reads
**OPTI**	fresh	50,000	1	77,750	95.1	0.26	43
			2	88,151	97.3	0.27	54
		10,000	1	87,362	98.8	0.27	49
			2	93,527	98.6	0.28	48
	cryopreserved	10,000	1	72,421	97	0.27	49
			2	78,125	97.6	0.28	51
	flash frozen	50,000	1	34,134	63.1	0.19	41
			2	41,840	71.6	0.18	29
**Omni + NP**	flash frozen	50,000	1	43,929	85.5	0.19	11
			2	44,280	88.6	0.2	6

OPTI-ATAC shows similar results between fresh and cryopreserved samples. Omni-ATAC protocol with  nuclear preparation (NP) on 50,000 flash frozen MCF7 cells is shown for comparison.

### ATAC protocol optimization for frozen cells

**Figure 2:**
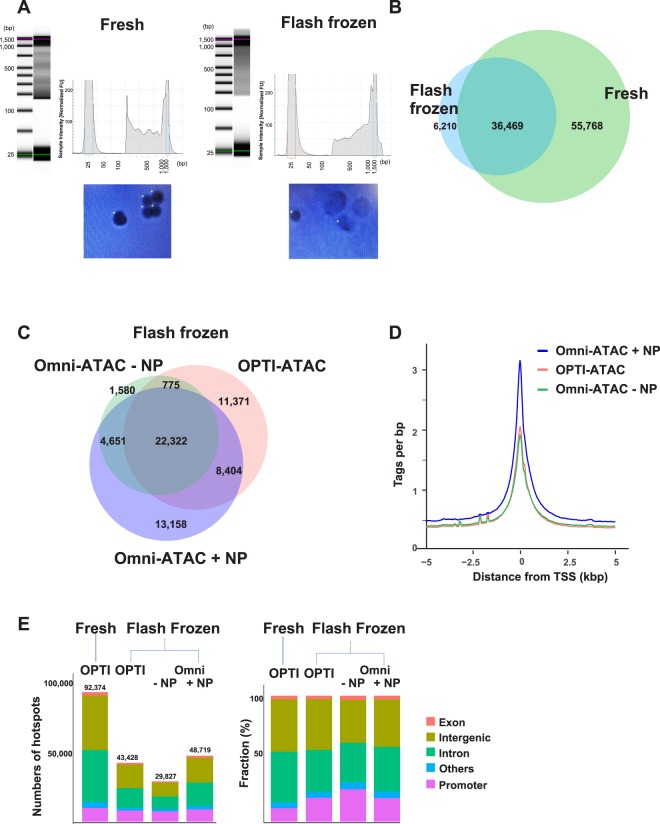
Testing ATAC-seq protocols using flash frozen MCF7 cells. (**A**) Top image: Bioanalyzer trace from OPTI-ATAC-seq libraries comparing fresh and flash frozen 50,000 MCF7 cells. DNA ladder is imaged on the first row for each set and the nucleosome phasing pattern is represented graphically on the right of each set. The nucleosome phasing pattern is not evident in flash frozen cells as opposed to fresh cells. Bottom: Nuclear morphology evaluated by trypan-blue staining shows intact nuclei in fresh cells, and distorted nuclei in flash frozen samples. The pictures were taken from display of TC20^TM^ automated cell counter (Bio-Rad, Hercules, CA, U.S.A) and yellow dots represents electronically counted cells. (**B**) Venn diagram comparing total number of hot spots from 50,000 fresh (green) and flash frozen (blue) MCF7 cells in merged replicates. (**C**) Venn diagram comparing ATAC-seq using the same number of flash frozen cells subjected to OPTI-ATAC (red) and Omni ATAC with and without nuclear preparation (+NP, −NP, blue and green, respectively). The data represents merged replicates. (**D**) Histogram of tag count enrichment reads at TSS ± 5 kb from merged replicates as above. (**E**) Distribution of differentially enriched genomic sites comparing OPTI-ATAC on fresh cells to OPTI-ATAC and Omni protocols on flash frozen cells from Venn diagram. Left bar graph represents the number of hot spots and the fractions are shown on the right. Genomic sites are represented as follows: exons (red), intergenic regions (light green), introns (dark green), promoters (magenta) and others (blue).

The lack of suitable methods for chromatin landscape analysis in stored samples has impaired its application to clinical samples, which are routinely fixed in formalin or flash frozen. Although formalin-fixed samples digested with DNase I are reported to provide some useful information^20^, the signal-to-noise ratio is low and the quality of the data is not suitable for genome-wide analysis. Unfortunately, frozen samples processed using the OPTI-ATAC protocol showed distorted nuclei and did not resulted in high quality data (<50% hot spot count compared to fresh cells) (Fig. 2A,B). Thus, we tested other ATAC-seq protocols using MCF7 cells flash frozen in liquid nitrogen.

Previous published studies suggested that the nuclear preparation step is not always necessary for open chromatin region analysis using flash frozen samples^17,21^. Thus, we performed the Omni-ATAC protocol with and without a nuclear preparation step (+/− NP). More peaks, a higher PRC value, and a higher signal-to-noise ratio was observed when the NP step was included (Fig. 2C). These data indicate that a nuclear preparation step is required for ATAC performed on flash frozen MCF7 cells. When comparing Omni-ATAC + NP with OPTI-ATAC-NP, we observed that they generated similar hot spot numbers and distribution, although Omni-ATAC + NP had a higher signal-to-noise ratio and enrichment of tag counts at TSSs ± 5 kb (Fig. 2C–E). Omni-ATAC + NP may be the best protocol for flash frozen MCF7 cells, but the data quality is much lower than fresh cells (Fig. 2E, Table 1).

**Figure 3:**
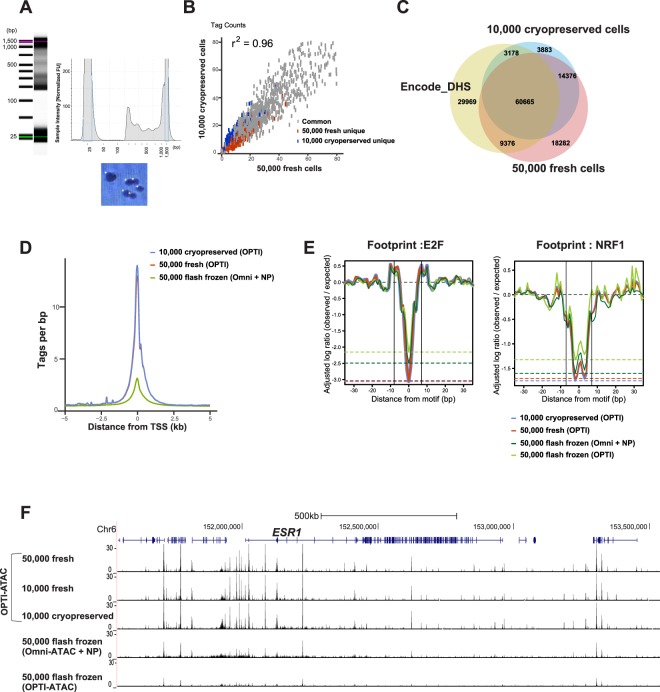
Analysis of OPTI-ATAC protocol using cryopreserved MCF7 cells. (**A**) Optimal nucleosome phasing pattern from Bioanalyzer (top) and nuclear morphology (bottom) are detected in as little as 10,000 cells. (**B**) Similar distribution of tag count between data of 50,000 fresh MCF7 and 4X transposase and the data of 10,000 cryopreserved MCF7 and 10X transposase. (**C**) Venn diagram confirms a large overlap between 50,000 fresh MCF7 with 4X transposase (red) and 10,000 cryopreserved MCF7 with 10X transposase (blue), both comparable to the DHS-seq data from ENCODE database for MCF7 cells (yellow). (**D**) Histogram of enrichment at TSS ± 5 kb confirms similarity between results obtained by OPTI-ATAC from fresh (red) and cryopreserved (blue) samples. Omni-ATAC from flash frozen cells with nuclear preparation (green) is presented for comparison. (**E**) E2F and NRF1 footprint. Log ratio of observed versus expected tag count was adjusted by the baseline of each data set and plotted as distance of binding motif. Similar results were detected using 50,000 fresh (red) and 10,000 cryopreserved cells (blue). Omni-ATAC (dark green) and OPTI-ATAC data (light green) data from flash frozen cells is presented for comparison. The two vertical black lines indicate the boundaries of the motifs. (**F**) Sequence track surrounding *ESR1* gene locus from UCSC browser on OPTI-ATAC from 10,000, 50,000 fresh, and 10,000 cryopreserved cells, and Omni-ATAC on 50,000 flash frozen cells. The 2 lower lanes show that flash frozen cells have lower signal and high noise above background. These data confirmed that cryopreservation can generate high quality data.

We then tested whether freezing cells via a different method could improve the ATAC-seq data quality. We stored cells by slowly freezing them in DMEM supplemented with 10% Dimethyl sulfoxide (DMSO) and 50% serum, which should protect against ice crystal formation and subsequent cell damage, hereafter referred to as cryopreservation. This approach produced excellent results and high-quality data comparable to fresh cells (Fig. 3). OPTI-ATAC using 10,000 cryopreserved cells produced similar total hot spot counts to the results obtained from 50,000 fresh cells, and to DNase-seq data from the ENCODE database, which was performed on millions of fresh MCF7 cells (ibid). Furthermore, similar genomic distribution and signal-to-noise ratio were observed (Supplementary Fig. 5A and Table 1).

Next, we performed digital footprinting analysis and detected footprints for E2F and Nuclear Respiratory Factor 1 (NRF1), key transcription factors associated with breast cancer progression and response to therapies^22–24^. High quality footprint data was generated from 10,000 cryopreserved and 50,000 fresh cells (Fig. 3E). An example UCSC genome browser screenshot from the *ESR1* gene locus is presented in Fig. 3F to confirm the open chromatin pattern using OPTI-ATAC protocol on 10,000 and 50,000 fresh as well as 10,000 cryopreserved cells. OPTI-ATAC and Omni-ATAC protocols using 50,000 flash frozen cells produced tracks with smaller peaks and higher background.

We further optimized the OPTI-ATAC protocol by increasing the concentration of transposase from 2X to 4X and 10X. Higher transposase concentrations significantly improved total hot spot numbers and signal-to-noise ratios. Furthermore, we obtained similar hot spot genomic distributions compared to ENCODE DNase-seq data (Supplementary Fig. 5A–C). When comparing results obtained using OPTI-ATAC and Omni-ATAC on 10,000 cryopreserved MCF7 cells, we determined that they generated a similar hot spot distribution (Supplementary Fig. 5D). However, OPTI-ATAC had a higher TSS score (Supplementary Fig. 5E). As OPTI-ATAC is a simpler procedure that minimizes DNA sample loss and is adaptable to small cell numbers, we concluded that the OPTI-ATAC protocol is ideal for generating high quality of data from as few as 10,000 cryopreserved human breast cancer cells.

We also confirmed that OPTI-ATAC was suitable for other cryopreserved breast cancer cell lines, T47D and ZR75-1 (Supplementary Fig. 6). Thus, the OPTI-ATAC protocol allows for high-quality genome-wide open chromatin site analysis using cryopreserved cells.

### Adaptation of the ATAC protocol for analysis of mouse mammary gland

Finally, we applied our experience optimizing ATAC for breast cancer cell lines to fresh, flash frozen, or cryopreserved tissue samples. Specifically, we used mouse mammary gland, with the ultimate aim to adapt this protocol to human normal and diseased breast tissues. Flash freezing is one of the current methods for sample storage in the clinic, and Omni-ATAC was previously reported to be suitable for flash frozen mouse and human tissues^17^. Therefore, we tested the Omni-ATAC protocol using flash frozen mouse mammary tissue and introduced several different detergent combinations and nuclear preparation steps (Supplementary Fig. 7). Unfortunately, none of these protocols, including OPTI-ATAC, which we developed for cryopreserved human breast cancer cells, gave sufficiently high-quality data using 50,000 nuclei isolated from mouse mammary gland (Supplementary Fig. 8 and Supplementary Table II).

**Figure 4:**
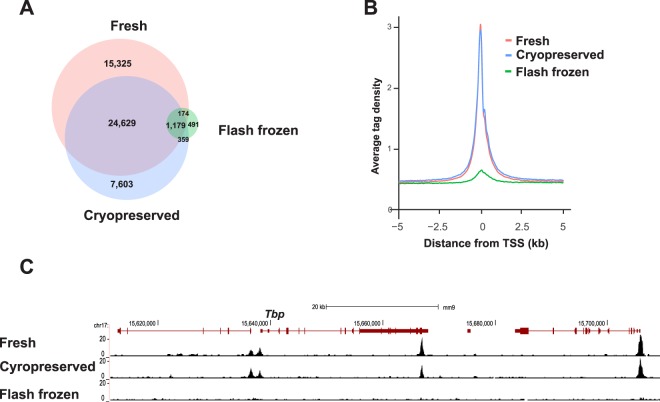
Comparison of fresh to flash frozen and cryopreserved mouse mammary tissues using 50,000 isolated nuclei and Omni-ATAC protocol. (**A**) Venn diagram of hotspots data from 50,000 nuclei obtained using fresh (red), flash frozen (green) and cryopreserved (blue) tissues in 1X transposase reaction. (**B**) Histogram comparing the same samples above. (**C**) Sequence track of *Tbp* gene locus from UCSC browser comparing the results from 50,000 nuclei isolated from fresh, cryopreserved and flash frozen tissues. These data confirmed that cryopreservation can generate high quality data comparable to fresh tissue samples.

As cryopreservation improved the ATAC data quality using breast cancer cells, we tested whether it could also improve the quality of the data generated using stored mouse mammary gland. Unfortunately, the OPTI-ATAC protocol using cryopreserved tissue did not significantly improve the results when compared to flash frozen tissue (Supplementary Fig. 8A). We then compared the results of 50,000 nuclei prepared from fresh, flash frozen and cryopreserved tissue using the previously published Omni-ATAC protocol. This protocol produced a well-defined nucleosome ladder, indicative of DNA fragments originally protected by an integer number of nucleosomes, that was comparable between fresh and cryopreserved tissues, as contrasted to flash frozen tissue (Supplementary Fig. 8B). Thus, we chose to perform the Omni-ATAC protocol on cryopreserved mouse mammary gland. Figure 4A,B show the results of Omni-ATAC protocol comparing 50,000 nuclei from fresh, flash frozen and cryopreserved tissues. Similar results were obtained from cryopreserved and fresh, including total hot spot count, distance from TSS, signal-to-noise ratio and genomic distribution (Fig. 4, Supplementary Table II). UCSC genome browser tracks near the mouse housekeeping gene locus, *Tbp*, confirm that cryopreserved tissue generated high quality data (Fig. 4C).

We further optimized the Omni-ATAC protocol to produce higher quality data. We found that removing the detergents (Digitonin and Tween 20, -D/T20) during the transposase reaction did not improve overall quality of the data (Supplementary Fig. 9A–D). However, increasing the transposase concentration doubled the hot spot count, increased the signal-to-noise ratio, and improved the overall data quality (PRC > 90%, Supplementary Fig. 9E–G and Supplementary Table II). Furthermore, 2X transposase concentration resulted in a higher fraction of open chromatin detected in intergenic and intronic regions (Supplementary Fig. 9H). Furthermore, cryopreserved samples had excellent reproducibility of separate experiments (Supplementary Fig. 10A).

We examined whether the data from cryopreserved tissue can be used to identify TF footprints in accessible chromatin regions. CCCTC-Binding Factor (CTCF) and E2F footprint depths were comparable in cryopreserved tissue between the 1X and 2X transposase concentrations, and was much improved compared to flash frozen tissue (Supplementary Fig. 10B). Overall, we conclude that cryopreservation is a viable storage method for both cells and tissues for future identification and analysis of open chromatin sites.

## Discussion

In this study, we demonstrated that ATAC-seq can generate high quality genome-wide chromatin landscape data from breast cancer cells and mammary tissue stored by cryopreservation that is comparable to the results obtained from fresh cells and tissue.

Cryopreservation is an effective strategy for structural preservation of most mammalian cell types and is widely applied to cell banking, including umbilical cord-derived blood cells and embryonic cells used for assisted reproduction. DMSO is frequently used as a cryoprotectant to prevent intra- and extracellular ice crystal formation that damages cellular structures, including chromatin. However, rapid thawing followed by cryoprotectant dilution prevents mechanical damage. Using this strategy, we cryopreserved different breast cancer cell types and found that ATAC-seq performed using only 10,000 cells yielded high quality data comparable to an equal number or even 50,000 fresh cells. The small cell numbers used in our studies is comparable to analysis of one or two fine needle aspiration biopsies^25^. This is applicable to future translational research because it can be used to evaluate the chromatin accessibility in early stage tumors and intraoperative samples without damage to tumor margins.

Cryopreservation of tissues is more difficult than other storage procedures because tissues are a mixture of various cell types and optimal cryopreservation strategies can vary between cell-types. We found that ATAC-seq can be performed on small samples of fresh tissue fragments and those frozen in DMSO-containing media, such as the commercially available BamBanker^26^. We confirmed that similarly cryopreserved human liver tissue in BamBanker generated high quality data (data not shown). However, OPTI-ATAC, which was best for human breast cancer cells, was not as successful as Omni-ATAC protocol for mouse mammary tissue. This could be because Omni-ATAC protocol requires multiple mild detergents compared to cultured cells, although longer incubation time is needed for tissue and nuclei preparation.

Unfortunately, flash frozen cells or tissue samples generated low-quality data regardless of the ATAC-seq protocol, compared to fresh and cryopreserved samples. Furthermore, inappropriate ATAC and storage protocols detected a lower open chromatin site fraction in non-promoter regions. These results indicate that storage and ATAC protocol optimization is essential for enhancer region detection, which is cell specific but can characterize disease status.

To select the best ATAC-seq protocol, we used several rigorous measurements of quality control. TSS score, recommended by the ENCODE consortium to estimate signal-to-noise ratio, is not sufficient to estimate the coverage of all potentially open chromatin sites in the genome in each assay. Thus, our laboratory developed a novel method for quality control, PRC, a powerful analysis of coverage depth for all potentially open hotspots in the genome, including those near TSS. Combining these two measurements provided excellent quality control for experiments performed on different days, and in comparing storage conditions, leading to selection of optimal ATAC protocol for cells and tissues.

In conclusion, our study provides critical information that will help researchers to optimize clinically-applicable ATAC-seq protocols and use stored tissues for genome-wide chromatin landscape analysis, transcriptional factors footprint, and, ultimately, disease-specific enhancer characterization. Selecting the correct freezing method is critical when using stored material for chromatin landscape analysis and should be considered in the development of future clinical trials. Our results will greatly expand the range of future translational research. Adapting ATAC-seq for stored clinical samples will lead to identification of epigenetic features and development of novel clinical targets not only for breast cancer, but also for many other diseases.

## Methods

### Cell lines

MCF7, T47D and ZR75-1 cells were obtained from ATCC (Manassas, VA), and cultured in 5% CO_2_ in a 37 °C incubator. MCF7 and T47D were maintained in Dulbecco’s Modified Eagle Medium (DMEM) with 4.5 g/l of D-glucose, and supplemented with 10% FBS, 2 mM L-glutamine, 1 mM sodium pyruvate, and 1% penicillin/streptomycin. ZR75-1 cells were maintained in RPMI 1640 containing the same supplements. Cells were prepared using three different methods:

#### Fresh cells

Cells were washed in phosphate-buffered saline (PBS), trypsinized, trypsin neutralized with culture medium, cells pelleted by centrifugation, and washed in PBS. Cells were counted, 50,000 cells were centrifuged at 3,000 rpm for 5 min at 4 °C and pellets were resuspended in cold cell lysis buffer followed by a nuclear preparation step^11,12^, as below.

#### Cryopreservation of cells

Cells were trypsinized, trypsin neutralized with culture media, spun down and the cell pellets were resuspended in slow freezing media (10% DMSO, 50% FBS, with 40% DMEM or RPMI 1640), transferred to an isopropyl alcohol chamber (Thermo Fisher Science, Waltham, MA), frozen slowly (−1 °C/minute) at −80 °C and stored for more than one month. To thaw, the vials were warmed for approximately 2 min in a 37 °C water bath, mixed with PBS (1:1) and centrifuged at 3,000 rpm for 5 min at 4 °C. The supernatant was removed, cell pellets were resuspended in cold PBS and counted. 10,000–50,000 cells were transferred to individual tubes, centrifuged at 3,000 rpm for 5 min at 4 °C, resuspended in cold cell lysis buffer.

#### Flash-frozen

The cells were washed with PBS, trypsinized, Trypsin neutralized in medium, counted, 50,000 cells were divided into individual vials and pelleted by centrifugation. The supernatant was removed, cells were flash-frozen in Eppendorf vials submerged in liquid nitrogen and stored in −80 °C for more than one month. The flash-frozen cell pellets were removed from −80 °C immediately and processed using the different ATAC protocols outlined below.

### Mouse mammary gland tissue

C57BL/6 J female mice (four to five months old) from the National Institutes of Health animal facility were sacrificed by CO_2_ inhalation, mammary glands were dissected and processed as described below. All experiments were approved by the ACUC (Animal Care and Use Committee) of the National Cancer Institute, National Institutes of Health and all methods were performed according to relevant guidelines and regulations from ACUC.

#### Fresh samples

Tissues were washed in ice-cold PBS containing protease inhibitors (Sigma, St. Louis. MO) and immediately processed for ATAC protocol described below.

#### Cryopreserved samples

Tissues were washed in ice-cold PBS containing protease inhibitors, cut into 4–5 mm diameter pieces, and several fragments were slowly frozen in 1–1.5 ml freezing media as described above. To thaw, vials were warmed for 2 min in a 37 °C water bath, diluted in PBS containing protease inhibitors, and the supernatant removed. The samples were minced by a razor blade on ice, followed by ATAC protocol as below.

#### Flash-frozen

Mammary glands were washed in PBS containing protease inhibitors, excess liquid removed on adsorbent tissue, cut into 4–5 mm size pieces, and three to five fragments were flash-frozen in liquid nitrogen and transferred to pre-chilled vials. The vials were stored in −80 °C for more than one month. The samples were removed from −80 °C, immediately minced by a razor blade on ice, and subjected to the ATAC protocol as below.

### Nuclear preparation of cell lines for ATAC protocol

The ATAC protocols^11,12^ were modified using the following changes: To prepare nuclei, 10,000–50,000 cells were pelleted and resuspended in ice-cold lysis buffer (20 μl for 10,000 cells, 25 µl for 25,000 cells, and 50 μl for 50,000 cells). We compared three different lysis buffers: original ATAC protocol^11^: 10 mM Tris-HCl pH 7.4, 10 mM NaCl, 3 mM MgCl_2_, 0.1% NP40; Takaku-ATAC protocol^18^: 10 mM PIPES pH 6.8, 100 mM NaCl, 300 mM sucrose, 3 mM MgCl_2_, 0.1% TritonX-100; and Omni-ATAC protocol^17^: 10 mM Tris-HCl pH 7.4, 10 mM NaCl, 3 mM MgCl_2_, 0.1% NP40, 0.1% Tween-20, 0.01% Digitonin. For the original and Takaku ATAC protocols, cells were incubated on ice for 5 min and spun at 2,500 rpm for 5 min at 4 °C. For Omni-ATAC, the cell pellets were resuspended with Omni-ATAC lysis buffer on ice (fresh and cryopreserved for 5 min, flash frozen for three minutes). ATAC-resuspension buffer (10 mM Tris-HCl pH 7.4, 10 mM NaCl, 3 mM MgCl_2_) containing 0.1% Tween 20 (0.5 ml for 10,000 cells, 1 ml for 50,000 cells) was added and the samples were centrifuged at 2,500 rpm at 4 °C (fresh and cryopreserved for 5 min, flash frozen cells for 10 min). The nuclei pellets were gently resuspended in transposase reaction mix, followed by transposase reaction.

### Nuclear preparation of tissue

The Omni-ATAC method for mouse mammary tissue was modified based on Corces *et al*.^17^. Approximately 100 mg of mouse mammary tissue was minced into ~2 mm fragments by a razor blade on ice, placed into a pre-chilled 1 ml Dounce homogenizer containing 1 ml of cold homogenization buffer (320 mM sucrose, 0.1 mM EDTA, 5 mM CaCl_2_, 3 mM Mg(Ac)_2_, 10 mM Tris-HCl pH 7.8, proteinase inhibitors, and 167 μM β-mercaptoethanol). 0.1% NP40 was added during the Nuclear Preparation step (+NP), or no detergents if NP was omitted (−NP). We also used 0.1% TritonX-100 as described in the OPTI-ATAC protocol. Tissues were homogenized by 10 strokes using a loose pestle, followed by 20–30 strokes with the tight pestle. Tissue homogenate (400 μl) were transferred to pre-chilled new 2 ml Lo-Bind Eppendorf tube and mixed with equal volume of 50% iodixanol in homogenization buffer to obtain a final of 25% iodixanol. 600 μl of 29% iodixanol in homogenization buffer was layered underneath the buffer containing the samples, with 600 μl of 35% iodixanol in same buffer was introduced below the 29% iodixanol. Nuclei were centrifuged at 4,000 rpm for 20 minutes at 4 °C in a swinging-bucket centrifuge. The nuclei in the layer formed between 29% and 35% iodixanol were collected into a new tube and counted by staining with trypan blue. 50,000 nuclei were transferred into a pre-chilled new tube containing 1 ml ATAC-resuspension buffer with 0.1% Tween-20 for Omni-ATAC or without detergent for OPTI-ATAC, and centrifuged at 2,500 rpm for 10 minutes at 4 °C. Supernatant was removed and the nuclei pellets was resuspended in transposase reaction mix described below.

### Transposase reaction (chromatin tagmentation)

Following the nuclei preparation, pellets were resuspended in respective reaction mixes with transposase (Illumina, San Francisco, CA) (Supplementary Table 3). We used 2x TD buffer (Illumina, San Francisco, CA) for the original, Takaku ATAC and OPTI-ATAC protocols, and 2x Omni-TD buffer (20 mM Tris-HCl pH7.6, 10 mM MgCl_2_, 20% Dimethyl Formamide in water for Omni-ATAC protocol. For experiments omitting nuclei preparation (−NP, +D/T20) in 50,000 flash frozen cell pellets, vials were removed from −80 °C and immediately resuspended in transpose reaction mix described in Supplementary Table III.

The transposase reaction was carried out for 30 minutes at 37 °C, in a shaker at 1,000 rpm. The samples were purified using MinElute PCR purification kit (Qiagen, Frederick, MD) and eluted into 10 μl of Elution Buffer. Samples were PCR-amplified using 1X NEBNext High-Fidelity PCR Master Mix (New England Biolabs, MA), 1.25 μM of custom Nextera PCR primers as described^11^, using the following PCR protocol: 5 min at 72 °C, 30 sec at 98 °C, followed by thermocycling (10 sec. 98 °C, 30 sec. 63 °C, 1 min 72 °C). After five amplification cycles, 5 μl aliquot was removed and added to 10 μl of the PCR mixture with SYBR Green I (Invitrogen, Carlsbad, CA) and amplified for 20 cycles to determine the number of additional cycles based on the cycle number that corresponds to a third of the maximum fluorescent intensity. Libraries were amplified for a total of 7–10 cycles. Subsequent sample purification and size selection (150–1000 bp) were performed using SPRI select beads (Beckman Coulter, Indianapolis, IN). Fragmented DNA was eluted in 25 µl 10 mM Tris-HCl, pH 8.0. The quality of the tagmented libraries was visualized by Agilent D1000 ScreenTape on 2200 TapeStation system (Agilent Technologies, Savage, DE).

### Sequencing and data processing

The samples were subjected to paired-end sequencing using 2 × 75-bp reads using the Illumina NextSeq High V2 at the National Cancer Institute Sequencing Facility, (Frederick, MD). The reads were trimmed *in silico* to remove adapter sequences, low-quality reads, and 50 bp length using Trimmomatic 0.30 software. The reads were aligned to human (hg19) or mouse (mm9) reference genome using Bowtie2 alignment tool. Mitochondrial reads were filtered for the subsequent analyses. DNase-seq data of MCF7 cells from GEO accession # GSE32970 was downloaded from the UCSC ENCODE database.

### Peak calling and replicate concordancy

All peak calling was performed using MACS2 v.2.1.1^27^ with *callpeak–format BAMPE* parameters for paired ended reads and *callpeak*-nomodel-shift-75-extsize 150 for single-ended reads. For peak-calling of ATAC-seq data, all forward strand reads were offset by +4 bp and all reverse strand reads were offset by -5 bp to represent the center of the transposon binding event^11^. For the replicates, we obtained the merged peak sets from pooled data as well as the sets of peaks from each individual replicate. We retained those peaks (referred in the text as hotspots) from pooled data that have at least 50% overlap in each replicate.

### Scatter plots, Venn diagrams, and Heatmaps

Scatter plots were generated to show the change in maximum tag densities in the sites between two replicates or conditions. Pearson correlations were calculated using R. For heatmaps, the total number of sequence reads under the 10 K base pair regions around the center of peaks has been extracted and normalized for the total number of reads in the sample (reads under the peak/10 million total reads) using annotatePeak.pl of the Homer Software Suite V. 4.10^28^. The heatmaps were generated using an in-house R script. For histograms, the data is presented as average read depth at each position in the surrounding 5,000 bp centered on RefSeq transcription start sites (TSS) determined using Homer (annotatePeaks.pl tss). The total tag number was normalized to 10 million reads. Area proportional Venn diagrams were generated to demonstrate the numbers of hot spots shared by two or three conditions using the Venneuler R statistical software package. The number of overlapped or unique peaks was determined using the software BEDtools suite v2.27.0^29^.

### Transcription Start Site (TSS) Enrichment score

TSS enrichment score is the ENCODE-recommended parameter to evaluate signal-to-noise ratio of open chromatin for ATAC- seq. The score was calculated by counting transposition events in 1 bp bins in the regions ±2,000 bp around all TSSs in hg19 RefSeq for human cells or mm9 RefSeq for mouse tissue samples.

### Percent Reference peak Coverage (PRC)

Percent Reference peak Coverage (PRC) is a rate of hotspots overlapping with common open chromatin sites of the respective species. It was developed to ensure compatible levels of digestion by tagmentation between experiments. To calibrate PRC, commonly represented hotspot sites were identified as reference peaks in human (hg19) and mouse (mm9) genomes. For human reference peaks, ENCODE narrowPeak definition files were downloaded from http://hgdownload.cse.ucsc.edu/goldenPath/hg19/encodeDCC/wgEncodeAwgDnaseUniform/. We collected 1,858 DNase I hypersensitivity sites consistently accessible (>97%) over 125 human cells available from ENCODE database which includes normal differentiated primary cells (n = 71), immortalized primary cells (n = 16), tumor-derived cell lines (n = 30), and multipotent and pluripotent progenitor cells (n = 8). For mouse reference peaks, ENCODE narrowPeak definition files (DNase I Hypersensitivity by Digital DNase I from ENCODE/University of Washington) from 133 samples were downloaded from the UCSC golden path web site: http://hgdownload.cse.ucsc.edu/goldenPath/mm9/encodeDCC/wgEncodeUwDnase/. A total of 8,587 DNase I peaks were identified as common to all 133 samples.

### Identification of transcription factor recognition motif sites

Footprint analysis was performed for E2F and NRF1 for MCF7 cells and mouse mammary gland tissue. We downloaded the position weight matrices of each transcription factor from the JASPAR data base^30^. Candidate sites for each motif were identified using FIMO^31^ (ver. 4.10.1) with p < 10^-4^ to scan the human (GRCh37/hg19) and mouse (NCBI37/mm9) reference genomes.

### Footprint plot analysis

The footprint deviation was calculated by the log2 ratio between the aggregate observed counts and the aggregated expected counts from genome-wide sets of sites matching the corresponding motif. The depth was calculated as the mean of the log2 ratio over the candidate FIMO motif regions of the transcription factors which are also in the open chromatin regions. The observed count profiles were generated by taking the aggregation of the raw cut counts over the cognate motif element which is bound by the transcription factors within ATAC-seq peaks. The profiles were generated as previously described^32^ by taking the average of DNA hexamer frequencies centered at each nucleotide position from the total raw cut counts in the samples^33^. To eliminate the sequencing bias due to non-uniquely mapped bases, we calculated hexamer frequencies using the obtained 3′ mappability information of k-mers using the mappability program available as part of the PeakSeq package^34^. To adjust for differences in the depth of sequencing between samples, read counts used in the calculation of both observed and expected counts were normalized to 100 million reads.

## Supplementary information

Supplementary materials
