# Supplementary material for: High Quality ATAC-Seq Data Recovered from Cryopreserved Breast Cell Lines and Tissue
Source: Sci Rep. 2019 Jan 24;9:516. doi: 10.1038/s41598-018-36927-7 (PMC6345852; doi:10.1038/s41598-018-36927-7)
Supplement: Supplementary file 1 — Supplementary materials [file 41598_2018_36927_MOESM1_ESM.pdf]

Supplementary Materials

High Quality ATAC-Seq Data Recovered from Cryopreserved Breast Cancer Cell Lines and Tissue

Authors:

Saori Fujiwara, Songjoon Baek, Lyuba Varticovski, Sohyoung Kim and Gordon L. Hager*

Affiliations:

Laboratory of Receptor Biology and Gene Expression, National Cancer Institute, National Institutes of Health, Bethesda, MD 20892, USA

***Correspondence:**

Address correspondence to:
Gordon L. Hager (hagaerg@exchange.nih.gov)
Laboratory of Receptor Biology and Gene Expression, National Cancer Institute, NIH, Bethesda, MD 20892, USA

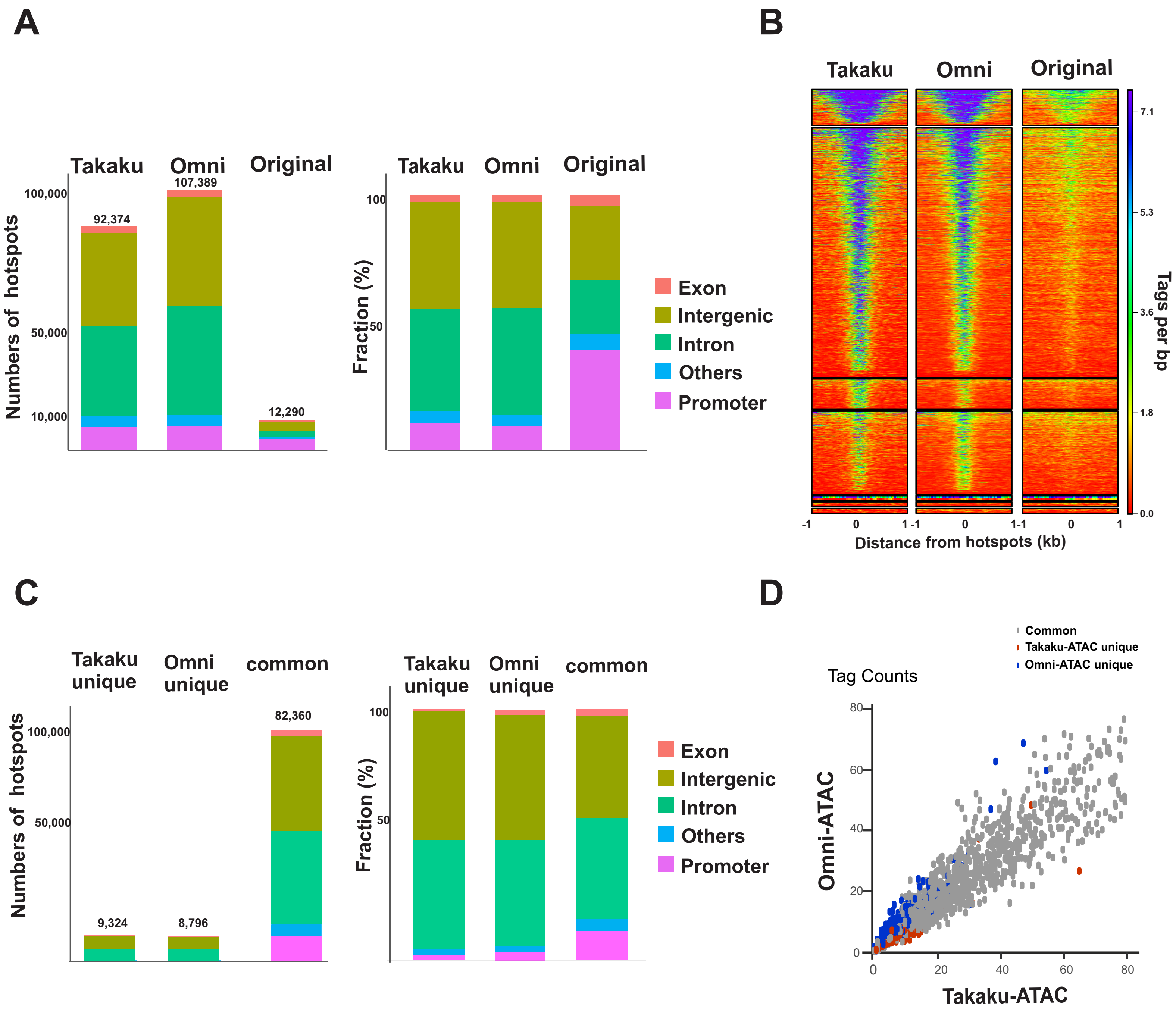

Supplementary Figure 1

A. Comparison of three ATAC protocols using 50,000 fresh MCF7 cells.
B. Distribution of differentially enriched sites using each of three ATAC protocols. Total hot spot count (left) and fraction of each genomic group (right).
C. Heatmap of the same samples as above within ± 1kb of the center of identified hot spots.
D. Distribution of differentially enriched genomic sites comparing the two best protocols using Takaku and Omni-ATAC protocols.
E. Scatterplot correlation of tag counts from ATAC performed using Takaku and Omni protocols shows similarity of the results.

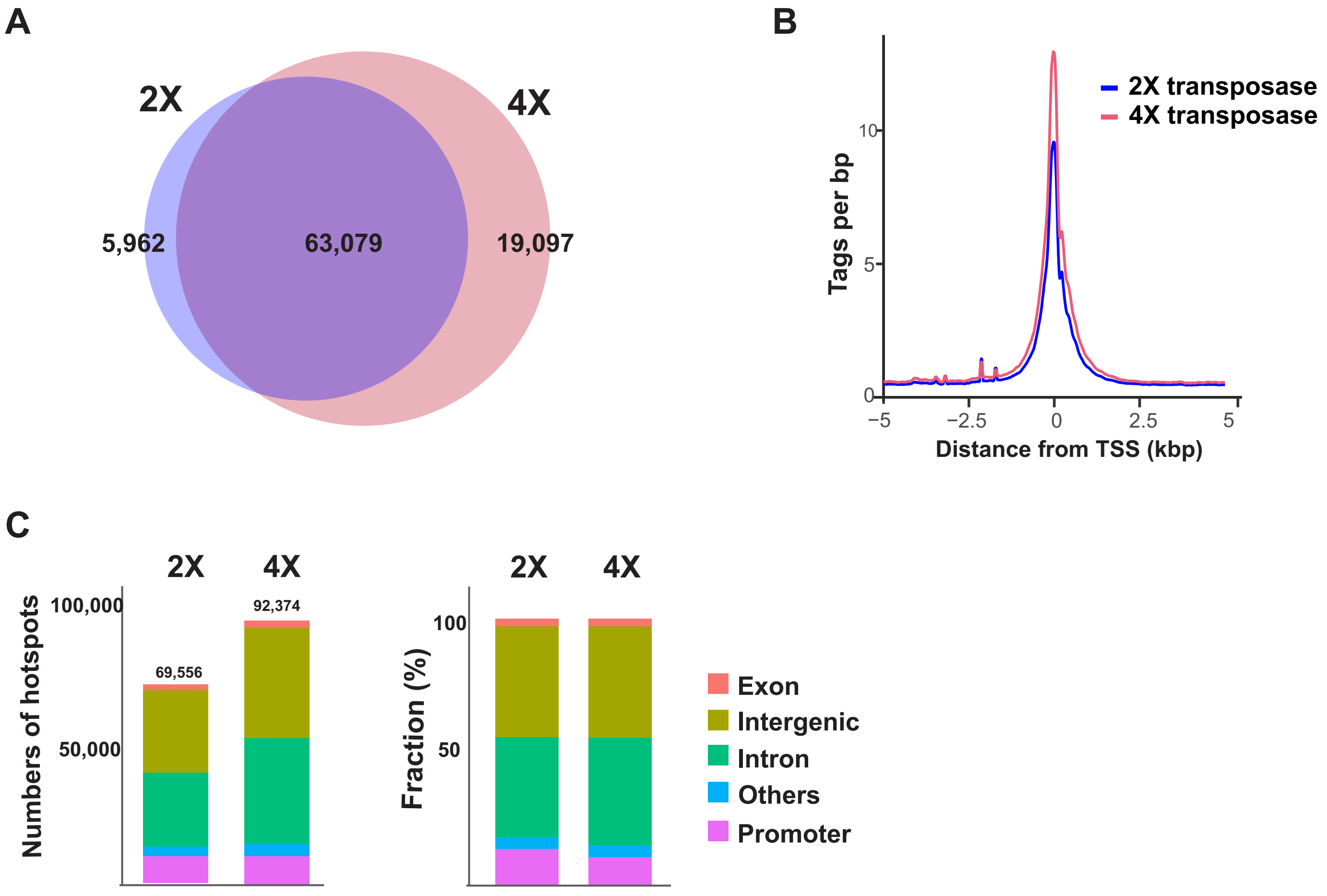

Supplementary Figure 2
Optimization of Takaku ATAC protocol (OPTI- ATAC).

A. Venn diagram shows overlap of hot spots from two computationally-merged ATAC-seq replicates using 2X and 4X transposase concentrations and 50,000 fresh MCF7 cells.
B. Enrichment of tag count reads at TSS ± 5 kb for merged replicates using 2X and 4X transposase concentration.
C. Distribution of differentially enriched genomic sites comparing 2X and 4X transposase concentration. Numbers of hot spots (left) and fraction of each genomic group(right).

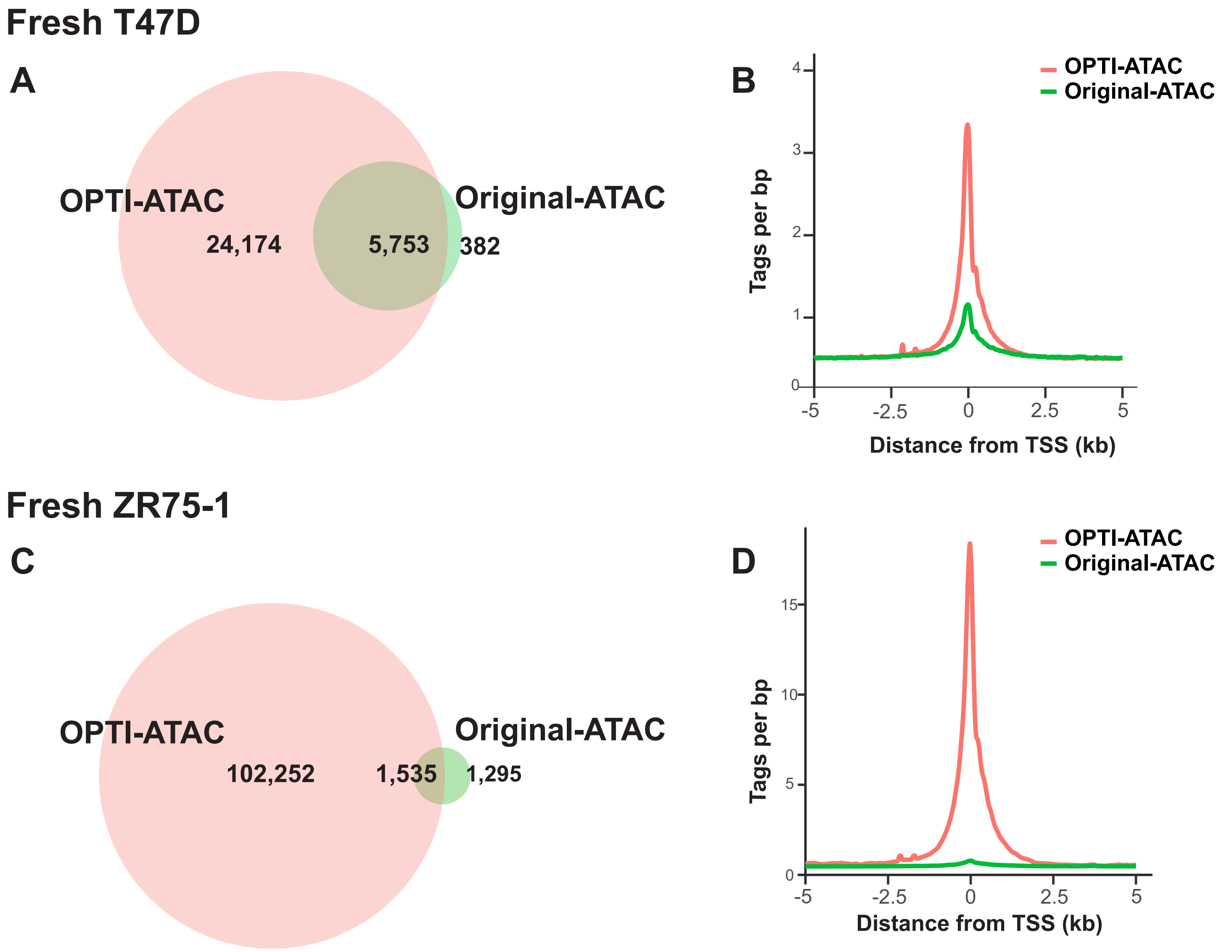

Supplementary Figure 3

Comparison of original and OPTI-ATAC protocols using 50,000 fresh cells: T47D (A, B) and ZR75-1 (C, D).

A. T47D: Venn diagram shows overlap of hot spots from two merged replicates.
B. T47D: Enrichment of tag count reads at TSS ± 5 kb for two merged replicates.
C. ZR75-1: Venn diagram shows overlap of hot spots from two merged replicates.
D. ZR75-1: Enrichment of tag count reads at TSS ± 5 kb two merged replicates.

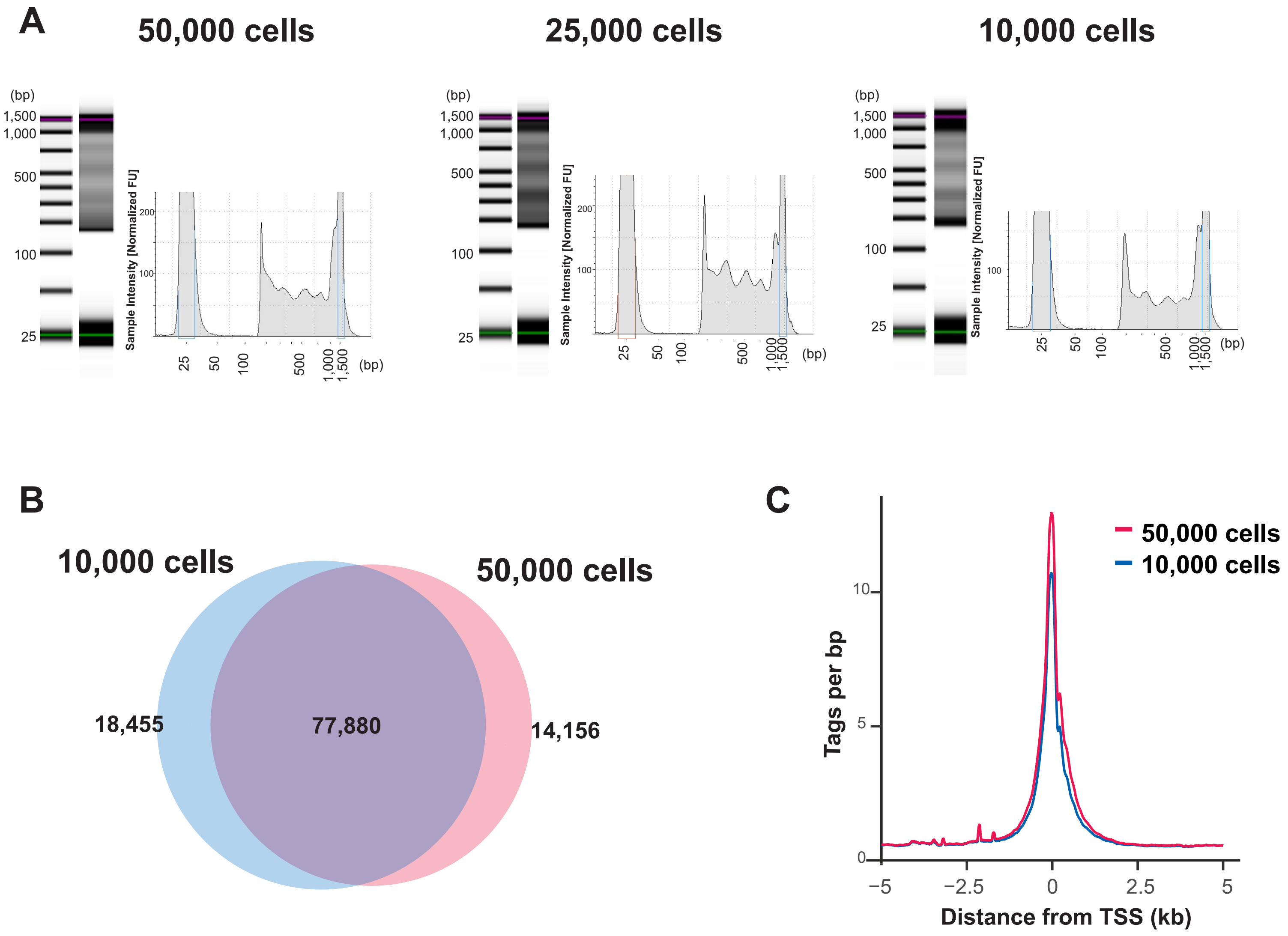

Supplementary Figure 4

OPTI-ATAC protocol results using smaller numbers of fresh MCF7 cells.

A. Bioanalyzer images of ATAC-seq libraries using 50,000, 25,000 and 10,000 cells. DNA ladder is imaged on the first row for each set. The nucleosome phasing pattern is evident in all samples and represented graphically on the right.
B. Venn diagram shows overlap of hot spots between 10,000 and 50,000 cells from merged replicates.
C. Histogram of tag count enrichment reads at TSS ± 5 kb from merged replicates as in Venn diagram.

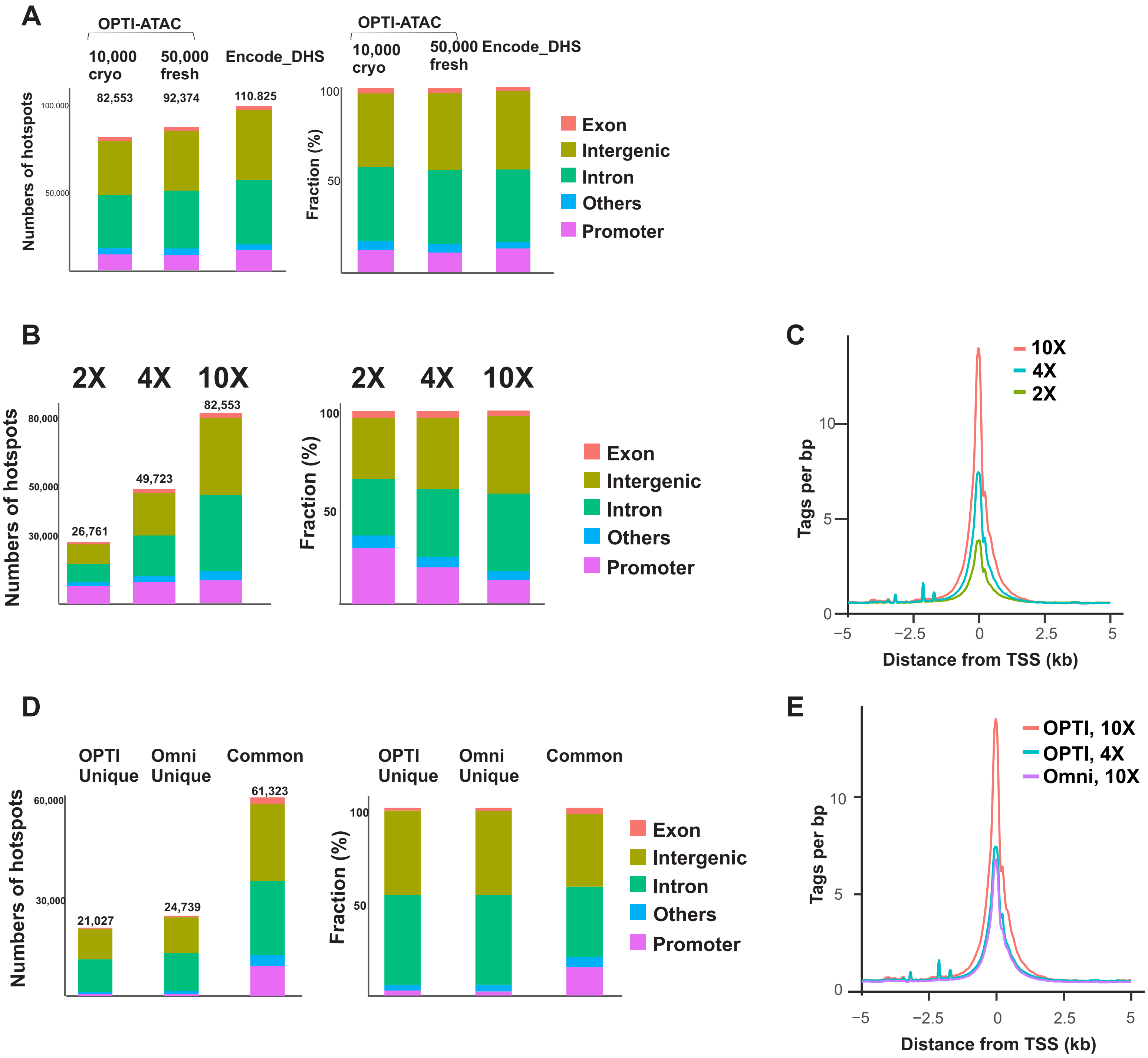

Supplementary Figure 5

Optimization of OPTI - ATAC protocol to 10,000 cryopreserved MCF7 cells.

A. Distribution of differentially enriched sites comparing OPTI - ATAC on 10,000 cryopreserved and 50,000 fresh MCF7 cells with ENCODE DHS-seq of MCF7 cells. Left bar graph represents numbers of hot spots and the fractions are shown on the right.
B. Distribution of differentially enriched sites comparing 2X, 4X and 10X transposase on 10,000 cryopreserved cells in 20ul reaction volume. Numbers of hot spots (left) and fraction of each genomic group (right).
C. Enrichment of tag count reads at TSS ± 5 kb for merged replicates showing best results from 10X transposase concentration.
D. Distribution of differentially enriched genomic sites comparing OPTI-ATAC to Omni-ATAC using 10,000 cryopreserved cells. Bar graphs represents total hot spots (left) and the fractions are shown on the right.
E. Enrichment of tag count reads at TSS ± 5 kb for merged replicates comparing OPTI-ATAC with 4X and 10X transposase concentration to Omni- ATAC using 10X transposase.

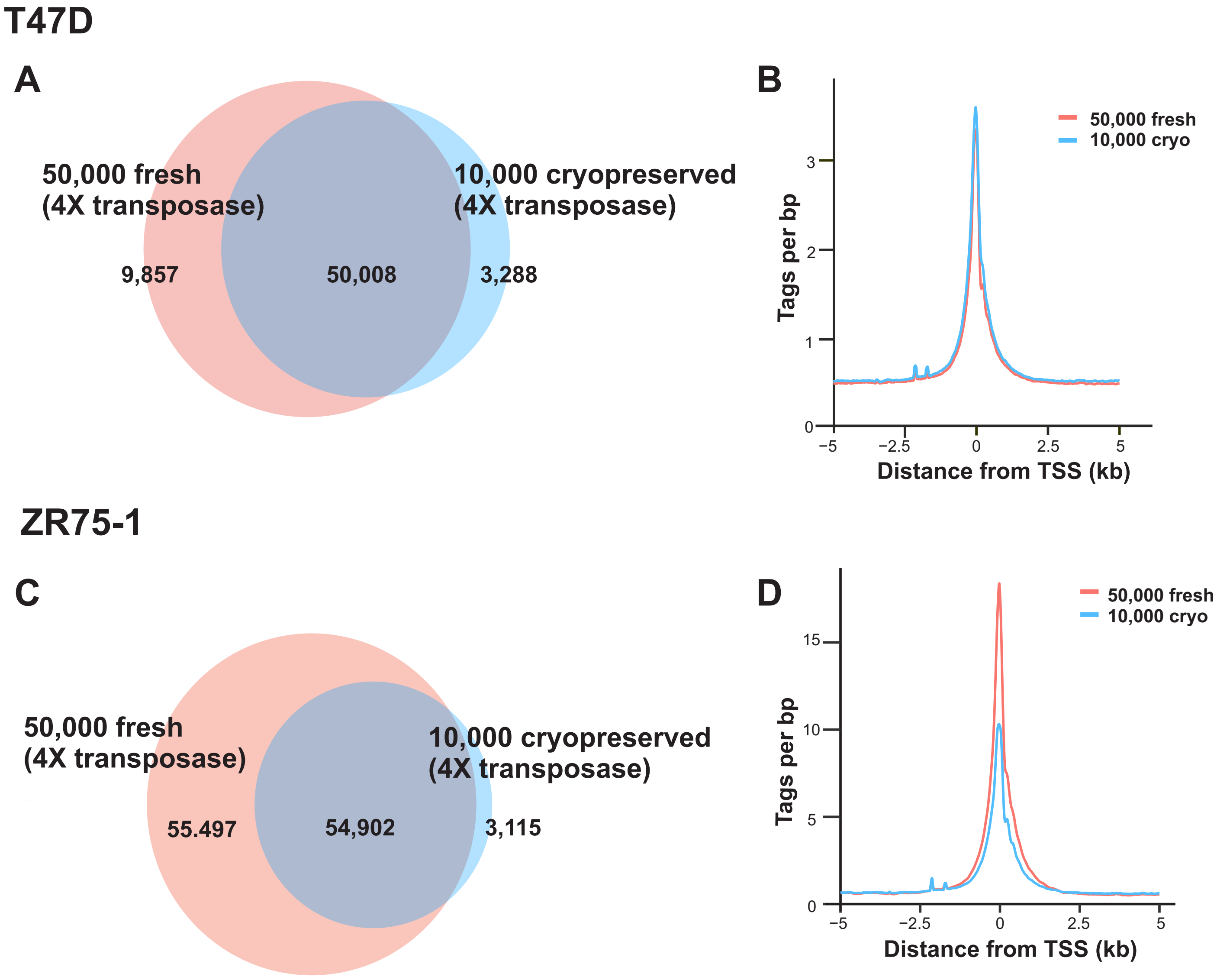

Supplementary Figure 6

Comparison of OPTI-ATAC protocols using 50,000 fresh cells and 10,000 cryopreserved cells: T47D (A, B) and ZR75-1 (C, D). In this figure 4X transposase was used for both 50,000 fresh cells (red) and 10,000 cryopreserved cells (blue).

A. Venn diagram confirms a large overlap between 50,000 fresh T47D with 4X transposase (red) and 10,000 cryopreserved T47D with 4X transposase (blue).
B. Enrichment of tag count reads at TSS ± 5 kb for two merged replicates of 50,000 fresh T47D with 4X transposase (red) and 10,000 cryopreserved T47D with 4X transposase (blue).
C. ZR75-1: Venn diagram shows overlap of hot spots from two merged replicates.
D. ZR75-1: Enrichment of tag count reads at TSS ± 5 kb two merged replicates.

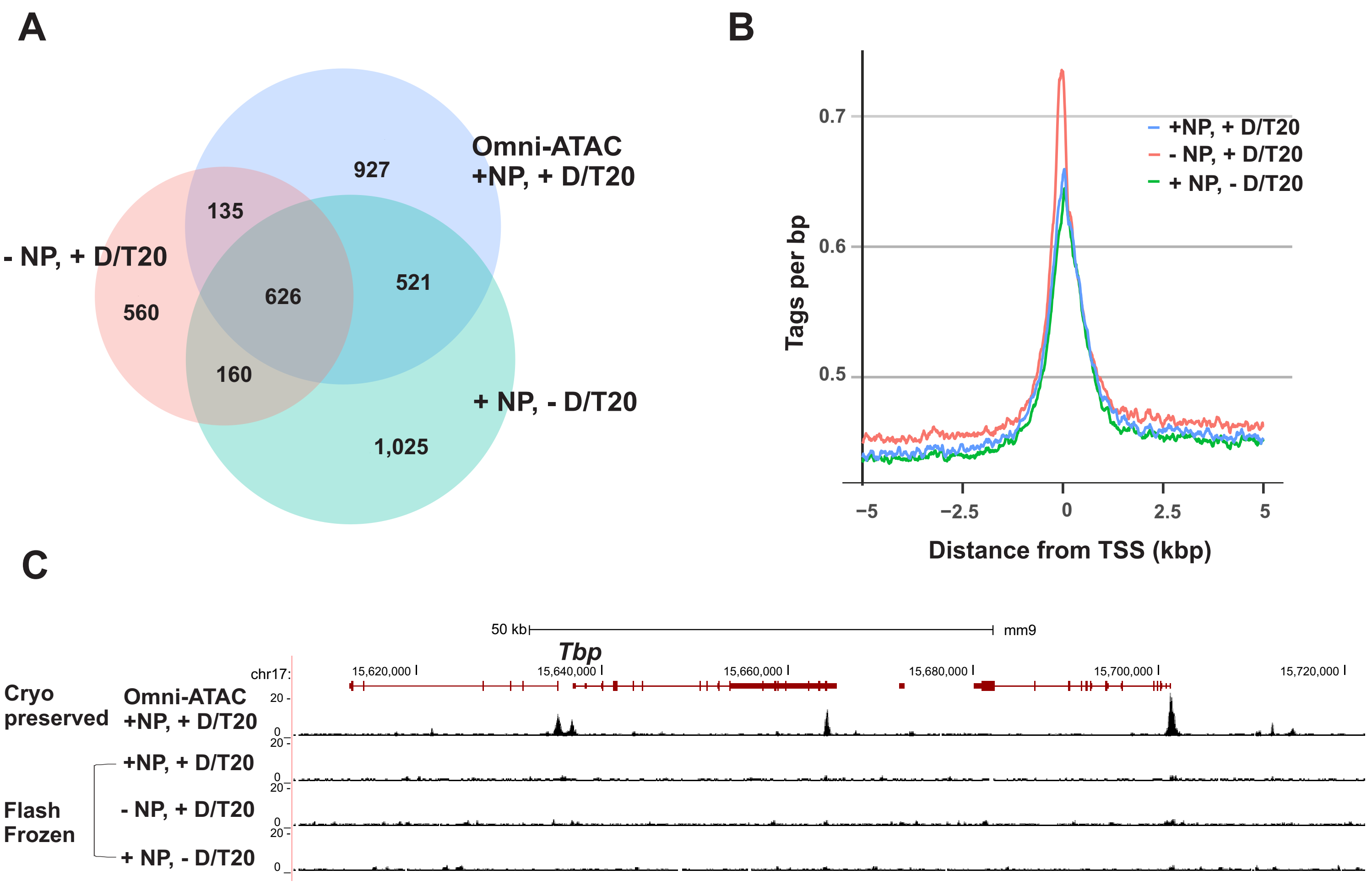

Supplementary Figure 7

Testing Omni- ATAC protocol on flash frozen mouse mammary tissues.

A. Venn diagram of hotspot count comparing original Omni- ATAC protocol with nuclear preparation and with two detergents in the transposase reaction, Digitonin and Tween-20 (+NP +D/T20, blue) to the same protocol without nuclear preparation (-NP + D/T20, Pink) and modified protocol with nuclear preparation but omitting the detergents in the transposase reaction (+NP, - D/T20, green).
B. Histogram comparing the same samples above.
C. Sequence track around *Tbp* gene locus from UCSC browser comparing cryopreserved samples tested by different protocols using flash frozen tissues as above. On the top lane, Omni-ATAC +NP+D/T20 from cryopreserved tissues is presented for comparison.

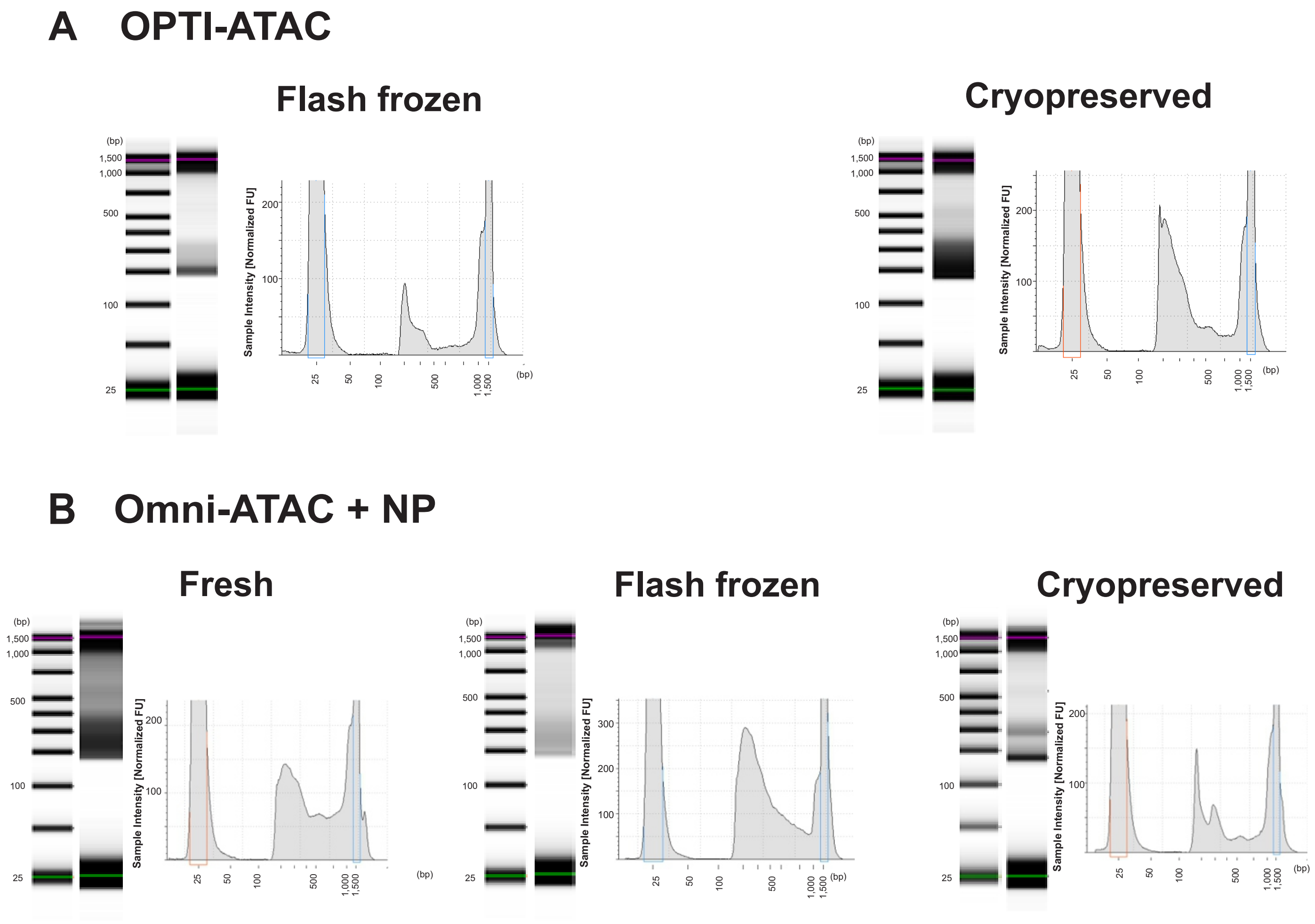

Supplementary Figure 8
Comparison of OPTI - ATAC and Omni- ATAC protocols using nuclear preparations from mouse mammary gland. Bioanalyzer images of nucleosome phasing pattern shows lack of clear nucleosome pattern in nuclei obtained for OPTI - ATAC from flash frozen or cryopreserved samples, and appropriate tagmentation from nuclei obtained from fresh or cryopreserved tissues using complete Omni-ATAC protocol which includes nuclear preparation step.

A. OPTI-ATAC using 50,000 nuclei isolated from flash frozen or cryopreserved tissues.
B. Omni-ATAC + NP using 50,000 nuclei isolated from fresh, flash frozen or cryopreserved tissues.

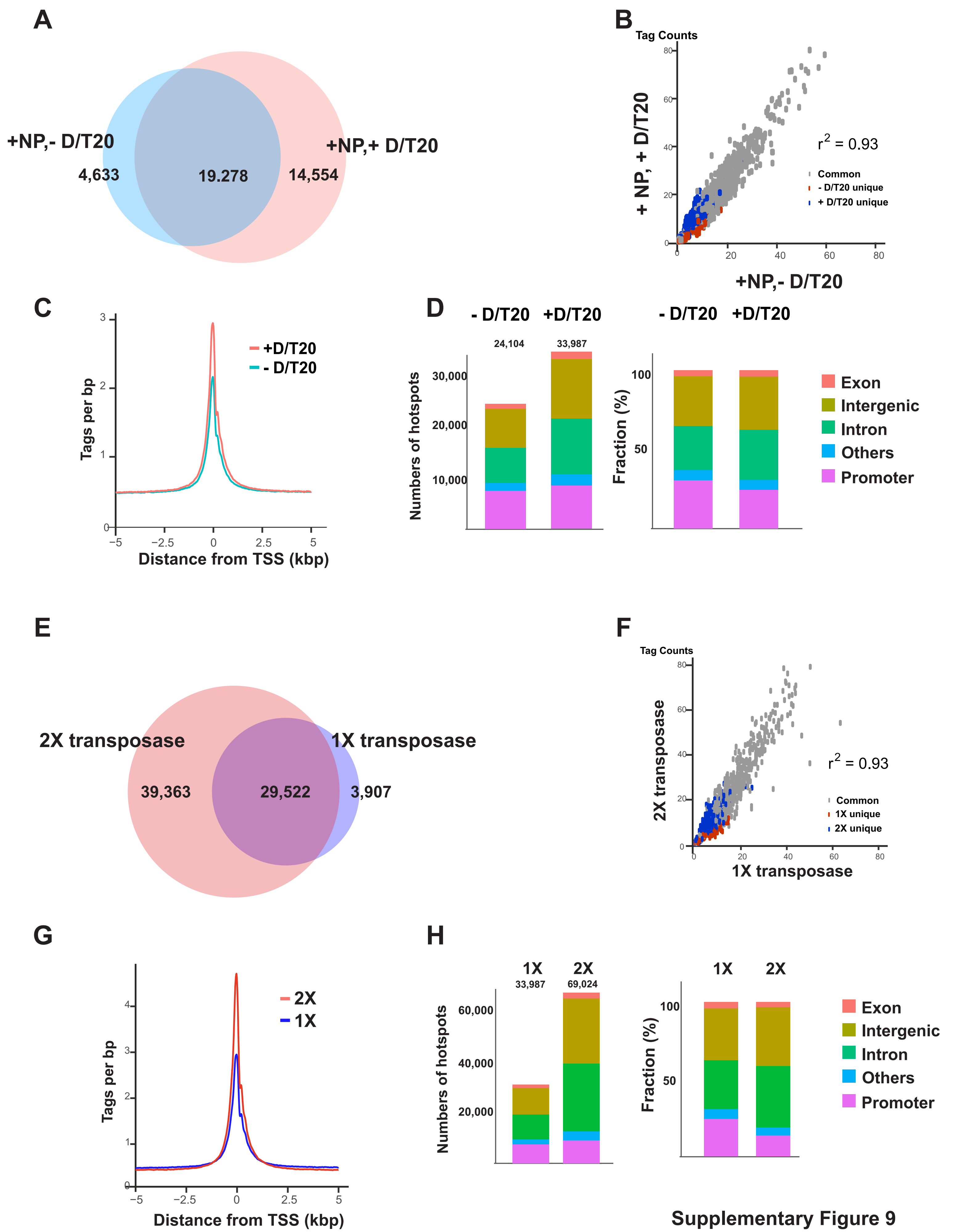

Supplementary Figure 9

Supplementary Figure 9

Adaptation of Omni-ATAC protocol to cryopreserved mouse mammary gland.

A. Venn diagram showing results from Omni-ATAC protocol with and without 2 detergents in the transposase reaction, +/- Digitonin and Tween20 (+/- D/T20).
B. Scatterplot comparing the results with and without Digitonin and Tween20 in the 1X transposase reaction.
C. Histogram showing significant improvement when detergents are added in the transposase reaction.
D. Distribution of hotspots in differentially enriched genomic sites comparing transposase reaction with and without detergents. Bar graphs represents total hot spots (left) and the fractions are shown on the right.
E. Venn diagram showing results from Omni-ATAC protocol with 2 detergents using 1X or 2X transposase concentration.
F. Scatterplot comparing 1X to 2X transposase concentration.
G. Distribution of hotspots comparing 1X to 2X transposase. Bar graphs on the left represent number of hot spots and the respective fractions are shown on the right.
H. Histogram showing significant improvement in using 2X transposase.

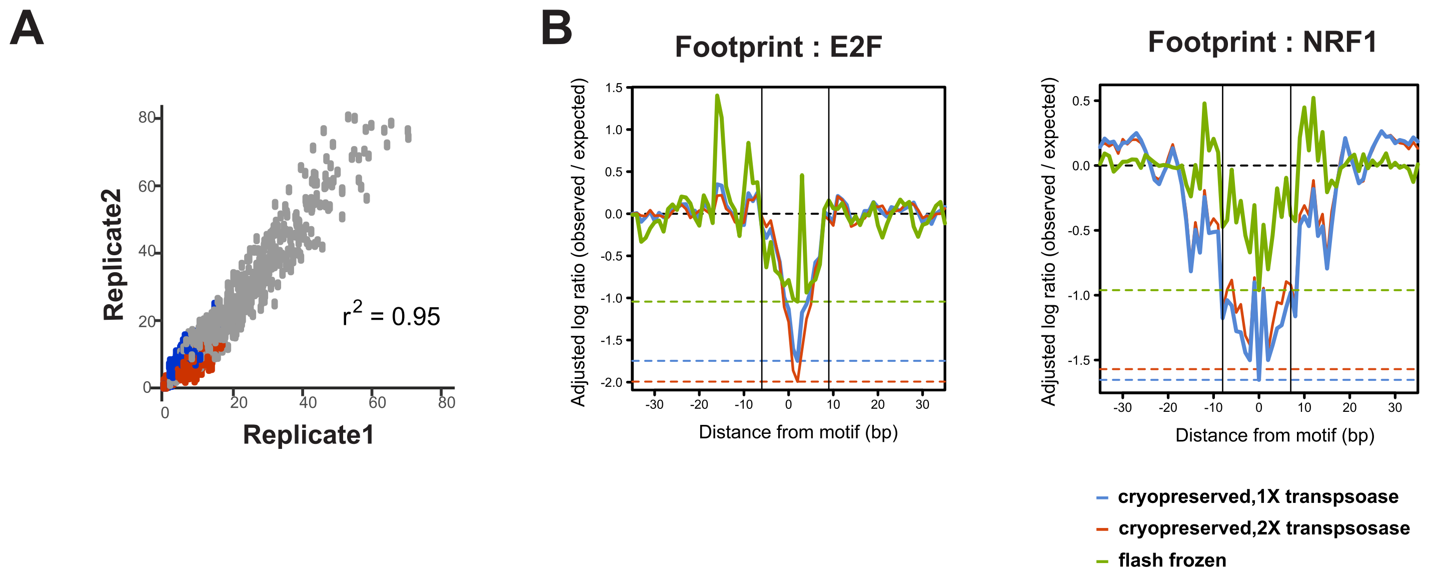

Supplementary Figure 10

A. Scatterplot of hotspots from two independent biological replicates using 50,000 nuclei from cryopreserved mouse mammary gland and Omni-ATAC protocol with 2X transposase. The data shows replicates concordance of r^2=0.95.
B. E2F and NRF1 footprints. Log ratio of observed versus expected tag count was adjusted by the baseline of each data set and plotted as distance to binding motif. High quality data was obtained from data using cryopreserved tissue from 1X (blue) and 2X (red) transposase. Omni-ATAC data from flash frozen tissues (green) is presented for comparison. The two vertical black lines indicate the boundaries of the motifs.

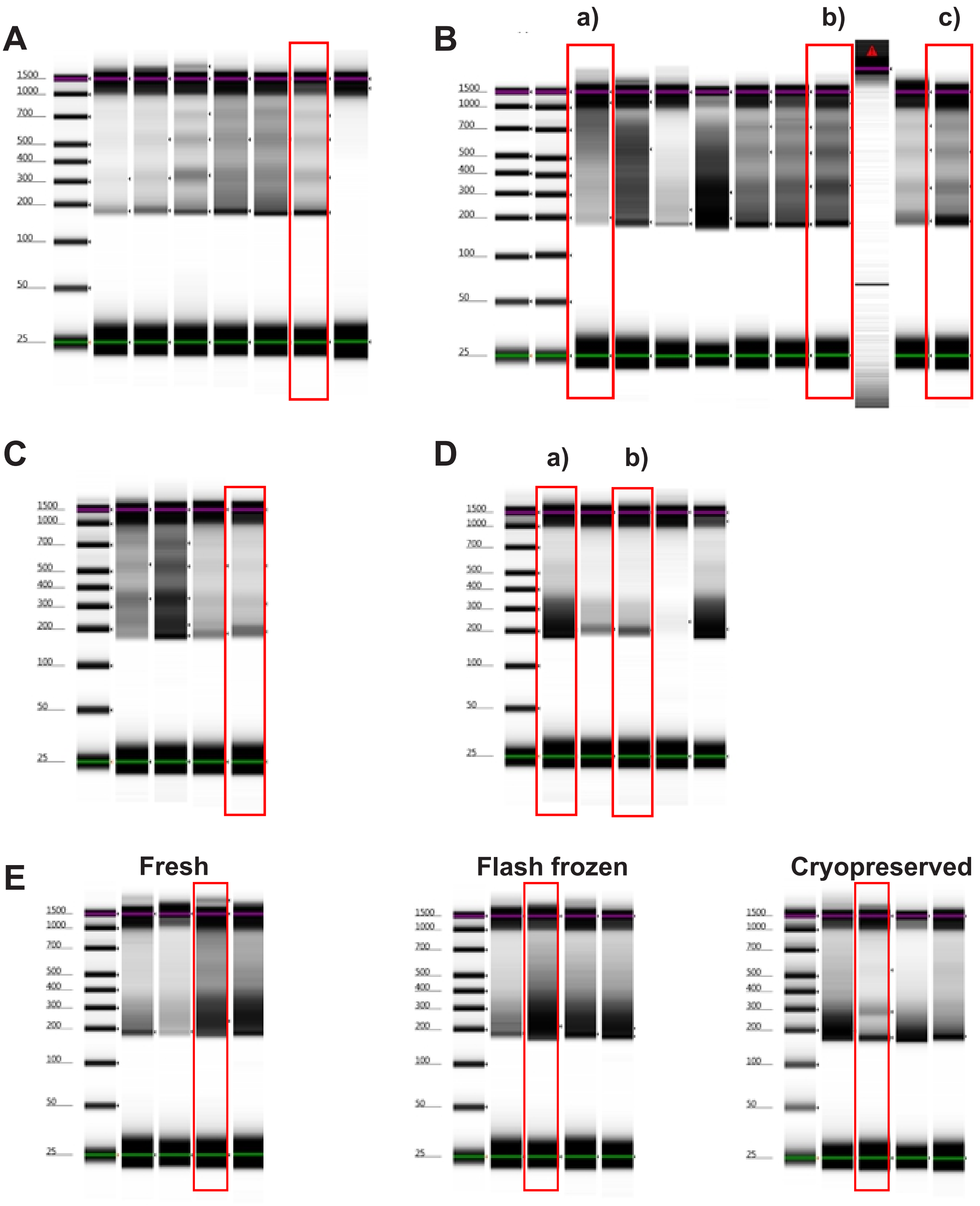

Supplementary Figure 11
Uncropped Bioanalyzer images of ATAC-seq libraries

A. For Fig. 2A and Supplementary Fig.4 : OPTI ATAC-seq libraries using 50,000 fresh MCF7 cells
B. a) For Fig.2A: OPTI ATAC-seq libraries using 50,000 flash frozen MCF7 cells
   b) For Supplementary Fig4: OPTI ATAC-seq libraries using 25,000 fresh MCF7 cells
   c) For Supplementary Fig4: OPTI ATAC-seq libraries using 10,000 fresh MCF7 cells
C. For Fig. 3A: OPTI ATAC-seq libraries using 10,000 cryopreserved MCF7 cells
D. For Supplementary Fig. 8A: OPTI ATAC-seq libraries using cryopreserved (a) and flash frozen (b) mouse mammary tissues
E. For Supplementary Fig. 8B: Omni ATAC-seq libraries using mouse mammary tissues

Supplementary Table I

Total hot spot count, PRC, TSS score and % mitochondrial reads for individual replicate of samples processed by ATAC-seq from human breast cancer cell lines.

Sample type	Storage method	Numbers of cells	ATAC method	Transposase concentration	Replicate	Numbers of hotspots	TSS score	PRC	Mitchondrial read(%)
MCF7	Fresh	50,000	OPTI-ATAC	4X	1	77,750	0.26	95.1	43.4
MCF7	Fresh	50,000	OPTI-ATAC	4X	2	88,151	0.27	97.3	53.5
MCF7	Fresh	10,000	OPTI-ATAC	10X	1	93,527	0.27	98.8	48.5
MCF7	Fresh	10,000	OPTI-ATAC	10X	2	87,362	0.28	98.7	47.9
MCF7	Cryopreserved	10,000	OPTI-ATAC	10X	1	72,421	0.27	97.0	49.4
MCF7	Cryopreserved	10,000	OPTI-ATAC	10X	2	78,125	0.28	97.6	50.9
MCF7	Cryopreserved	10,000	OPTI-ATAC	4X	1	47,352	0.26	92.9	58.8
MCF7	Cryopreserved	10,000	OPTI-ATAC	4X	2	37,584	0.24	86.8	57.3
MCF7	Cryopreserved	10,000	OPTI-ATAC	2X	1	19,171	0.21	58.2	43.5
MCF7	Cryopreserved	10,000	OPTI-ATAC	2X	2	25,749	0.23	72.0	50.6
MCF7	Cryopreserved	10,000	Omni-ATAC	10X	1	67,402	0.26	97.3	11.6
MCF7	Cryopreserved	10,000	Omni-ATAC	10X	2	59,914	0.25	97.0	10.8
MCF7	Flash Frozen	50,000	OPTI-ATAC	4X	1	34,134	0.19	63.1	40.9
MCF7	Flash Frozen	50,000	OPTI-ATAC	4X	2	41,840	0.18	71.6	28.6
MCF7	Flash Frozen	50,000	Omni-ATAC+NP	4X	1	43,929	0.20	88.6	10.8
MCF7	Flash Frozen	50,000	Omni-ATAC+NP	4X	2	44,289	0.19	85.5	5.8
MCF7	Flash Frozen	50,000	Omni-ATAC-NP	4X	1	30,297	0.18	67.8	26.4
MCF7	Flash Frozen	50,000	Omni-ATAC-NP	4X	2	19,348	0.18	62.1	24.6
T47D	Fresh	50,000	original-ATAC	4X	1	6,021	0.16	20.0	4.8
T47D	Fresh	50,000	original-ATAC	4X	2	3,618	0.14	8.7	3.2
T47D	Fresh	50,000	OPTI-ATAC	4X	1	19,170	0.21	69.6	17.0
T47D	Fresh	50,000	OPTI-ATAC	4X	2	33,486	0.24	88.4	22.6
T47D	Cryopreserved	10,000	OPTI-ATAC	4X	1	19,758	0.22	72.6	38.4
T47D	Cryopreserved	10,000	OPTI-ATAC	4X	2	21,860	0.22	73.5	33.5
ZR75-1	Fresh	50,000	original-ATAC	4X	1	2,728	0.13	4.3	2.5
ZR75-1	Fresh	50,000	original-ATAC	4X	2	2,153	0.12	2.1	2.6
ZR75-1	Fresh	50,000	OPTI-ATAC	4X	1	95,462	0.28	96.8	53.0
ZR75-1	Fresh	50,000	OPTI-ATAC	4X	2	95,407	0.28	96.9	50.5
ZR75-1	Cryopreserved	10,000	OPTI-ATAC	4X	1	48,953	0.25	91.9	55.2
ZR75-1	Cryopreserved	10,000	OPTI-ATAC	4X	2	50,040	0.26	90.5	58.8

Supplementary Table II
Total hot spot count, PRC, TSS score and % mitochondrial reads for individual replicate of samples processed by ATAC-seq from mouse mammary gland.

Mouse mammary gland (Omni-ATAC)								
Storage method	Omni ATAC method		Transposase concentration	Replicate	Numbers of hotspots	TSS score	PRC	Mitchondrial read(%)
	NP	D/T20						
Fresh	+	+	1X	1	48,337	0.23	92.7	6.8
Fresh	+	+	1X	2	10,697	0.19	61.9	2.3
Cyropreserved	+	-	1X	1	25,741	0.20	82.8	2.8
Cyropreserved	+	-	1X	2	16,966	0.18	70.9	1.6
Cyropreserved	+	+	1X	1	32,865	0.21	89.6	4.3
Cyropreserved	+	+	1X	2	24,774	0.20	84.3	3.3
Cyropreserved	+	+	2X	1	61,797	0.24	96.2	3.7
Cyropreserved	+	+	2X	2	62,556	0.24	96.4	3.2
Flash Frozen	+	+	1X	1	1,158	0.12	0.7	6.0
Flash Frozen	+	+	1X	2	2,206	0.12	3.4	1.7
Flash Frozen	+	-	1X	1	1,060	0.11	0.5	5.5
Flash Frozen	+	-	1X	2	2,783	0.12	3.3	1.2
Flash Frozen	-	+	1X	1	1,015	0.13	2.2	1.4
Flash Frozen	-	+	1X	2	1,132	0.13	4.5	2.3

Supplementary Table III

Transposase reaction mix for each sample.

Transposase reaction mix for the original,Takaku and OPTI-ATAC

cell/nuclei number	concentration	transposase (ul)	TD buffer (ul)	water (ul)
50,000	2X	5	25	20
50,000	4X	10	25	15
10,000	2X	0.4	10	9.6
10,000	4X	0.8	10	9.2
10,000	10X	2	10	8

Transposase reaction mix for Omni-ATAC

protocol	cell/nuclei number	concentration	transposase (ul)	Omni-TD buffer (ul)	PBS (ul)	water (ul)	1% Digitonin	10% Tween-20
+D/T20	50,000	4X	10	25	14	-	0.5	0.5
- D/T20	50,000	4X	10	25	14	1	-	-
+D/T20	10,000	10X	2	10	6.6	1	0.2	0.2
